# MicroRNA-506-3p inhibits colorectal cancer cell proliferation through targeting enhancer of zeste homologue 2

**DOI:** 10.1080/21655979.2021.1951930

**Published:** 2021-07-21

**Authors:** Liang Ai, Xiaojun Luo, Xiong Yan, Shan Jiang

**Affiliations:** a Department of Oncology, Chongqing Hospital of Traditional Chinese Medicine Chongqing City, China; b Department of Hepatobiliary and Pancreatic Tumor Center, Chongqing University Cancer Hospital, Chongqing City, China; c Department of Hepatobiliary Surgery, The First Affiliated Hospital of Chongqing Medical University, Chongqing City, China; d Department of Oncology, The First Affiliated Hospital of Chongqing Medical University, Chongqing City, China

**Keywords:** Mir-506-3p, colorectal cancer, enhancer of zeste homologue 2 (ezh2)

## Abstract

A large number of studies have shown that microRNA (miRNA) has an important relationship with the occurrence and development of colorectal cancer (CRC), but its specific molecular mechanism has not been fully elucidated. This study is to explore the influence of miR-506-3p on the malignant behavior of CRC and its underlying molecular mechanism. Our results show that miR-506-3p was lowly expressed and enhancer of zeste homologue 2 (EZH2) was highly expressed in CRC. Overexpressing miR-506-3p or silencing EZH2 inhibited CRC cell proliferation, migration and invasion and promoted apoptosis. Inhibiting miR-506-3p promoted CRC cell proliferation, migration and invasion but inhibited apoptosis. These impacts were reversed after co-transfecting si-EZH2. Further mechanism studies have shown that miR-506-3p can reduce EZH2 expression in CRC cells by binding to the 3ʹUTR end of EZH2. In summary, the results of this study show that miR-506-3p inhibited CRC progression through targeting EZH2 expression. This provides a new molecular target for the clinical treatment of CRC in the future.

## Graphical Abtstract

**Figure:**
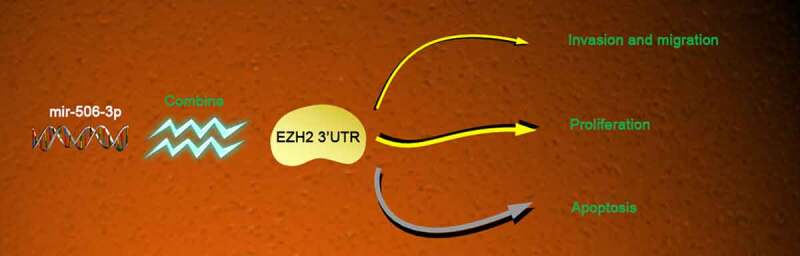

## 1.Introduction

Colorectal cancer (CRC), a universal malignant tumor in digestive system, seriously endangers human health. CRC occurrence and development are closely relative to heredity, lifestyle, and precancerous lesions [1,2]. It is estimated that CRC has become the third largest malignant tumor worldwide. About 2/3 CRC cases come from developed countries [3,4]. CRC incidence in Chinese population is on the rise, and the age of onset has been signally younger in recent years [5,6]. Based on previous studies, CRC is more common in people aged 40–70. CRC symptoms are atypical. At present, the methods of diagnosing colorectal cancer mainly rely on clinicopathological characteristics and imaging analysis [7]. In addition, more and more studies have shown that some cellular biomolecules (such as non-coding RNA and protein in peripheral blood) can be used as potential markers for the diagnosis of CRC [8,9]. However, it is necessary to seek more accurate early biomarkers of CRC to improve the survival rate of patients. The vast majority of CRC patients are already advanced or accompanied by metastasis at the time of diagnosis [10,11]. Therefore, there is an urgent need to improve the diagnosis and treatment of CRC.

MicroRNA (miRNA), highly conserved small non-coding single-stranded RNA molecules [12], regulates various cellular processes, like cell proliferation, metastasis, differentiation, along with metabolism through base pairing with target gene 3ʹ-UTR [13,14]. In addition, miRNAs are usually targeted by upstream circular RNA (circRNA) or long non-coding RNA (LncRNA) [15]. circRNA and LncRNA are RNA transcripts of miRNA recognition element (MRE), which act as competitive inhibitors of miRNA in the body and function by competing with endogenous mRNA for the binding site of miRNA. In reference to increasing studies, the abnormal function of miRNA affects CRC development and metastasis [16,17]. In neuroblastoma [18], glioma [19] and ovarian cancer [20] and others, down-regulating miR-506-3p is related to cancer cell proliferation, migration and apoptosis, which indicates that it is a tumor suppressor. But specific miR-506-3p mechanism in CRC proliferation and migration is still not clear.

EZH2, a histone lysine N-methyltransferase, regulates DNA methylation and inhibits RNA transcription [21,22]. It was highly expressed in various tumors including CRC [23], regulating CRC growth and metastasis [24]. Tumor suppressor miRNAs through directly targeting EZH2, inhibit CRC progression include miR-144 and miR-214 [25,26]. It has also been reported that EZH2 expression in CRC patients, links with greater lymph node metastasis potential together with lower overall survival rate [27]. Inhibiting EZH2 may become a potential target for treating CRC. Whether EZH2 expression is relative to miR-506-3p level in CRC has not been determined.

This work analyzed miR-506-3p and EZH2 expression in CRC tissues to explore the role of miR-506-3p and EZH2 in CRC progression, revealing that miR-506-3p acts pivotally in CRC cell growth and migration, together with confirming that EZH2 is miR-506-3p target. miR-506-3p/EZH2 signal transduction may be a potential strategy for treating CRC.

## 2. Patients & methods

### 2.1 Clinical specimens

From 2016 to 2018, CRC tissues and similar normal tissues from 30 patients who received CRC treatment in First Affiliated Hospital of Chongqing Medical University, all experimental procedures got Ethics board approval of the First Affiliated Hospital of Chongqing Medical University.

### 2.2 Cell culture

Human normal colorectal epithelial cells FHC and CRC cell lines (HCT-116, HT-29 and HCT-8) purchased from ATCC were cultured in modified Eagle’s medium (Carlsbad, U.S.) supplemented with 10% FBS (Invitrogen), penicillin (100 U/ml, Invitrogen) and streptomycin (100 μg/ml, Invitrogen) at 37°C, in a humid environment with 5% CO2.

### 2.3 Cell transfection

Cell transfection was performed as previously described [28]. GenePharma has synthesized miR-506-3p-mimic (5ʹUAAGGCACCCUUCUGAGUAGA-3ʹ), miR-506-3p-inhibitor (5ʹ-UCUACUCAGAAGGGUGCCUUA-3ʹ) and corresponding miR-NC (China), si-NC corresponding to small interfering RNA that targets EZH2 (si-EZH2) purchased from RiboBio Company (China). Based on the kit instructions, these reagents were transfected into HT-29 and HCT-8 cells with Liposome 3000 reagent (Invitrogen), the collected cells for subsequent experiments 72 h after transfection.

### 2.4 Real-time quantitative PCR (RT-qPCR)

Perform qRT-PCR as described previously [29]. After total RNA was obtained from the cells via TRIzol (Invitrogen), the cDNA was subjected to reverse transcription via PrimeScript RT kit (TaKaRa) based on instructions. Later, SYBR Green PCR master mix (TaKaRa) and Applied Bio-systems 7500 fast real-time RCR system (Applied Biosystems, U.S.) were employed for RT-qPCR analysis. Data from three independent experiments were standardized with GAPDH and U6 as the analysis standardization. Using 2−∆∆Ct relative gene expression level was calculated, and the primer sequences are in the following:

GAPDH: F: 5ʹ- ATGGGGAAGGTGAAGGTCG-3ʹ;

R: 5ʹ- TTACTCCTTGGAGGCCATGTG-3ʹ;

U6: F: 5ʹ- CTCGCTTCGGCAGCACATATACT-3ʹ;

R: 5ʹ- ACGCTTCACGAATTTGCGTGTC-3ʹ;

miR-506-3p: F: 5ʹ-GCCACCACCATCAGCCATAC-3ʹ;

R: 5ʹ-GCACATTACTCTACTCAGAAGGG-3ʹ;

EZH2: F: 5ʹ-CCCTGACCTCTGTCTTACTTGTGGA-3ʹ;

R: 5ʹ-ACGTCAGATGGTGCCAGCAATA-3ʹ;

### 2.5 Cell proliferation experiment

Cell proliferation measurement was done via CCK-8 kit (CK-04, Dojindo), later 2,000 HT-29 or HCT-8 cells were seeded into 96-well plates, and 10% CCK-8 medium was added to each well at 0 h, 24 h, 48 h, and 72 h, to incubate for 2 hours. OD value detection at 450 nm was done via enzyme-labeled instruments (EXL800, BioTek Instruments).

### 2.6 Western blot

The cells were lysed in RIPA lysis buffer (CWBIO, China). After quantification via BCA Protein Assay Kit (Sigma-Aldrich), 40 μg protein sample received electrophoresis on 15% SDS-PAGE, later was transferred to PVDF films (Bio-Rad Laboratories, Inc.) that were blocked with 5% skimmed milk to combine with the primary antibodies GAPDH (1:3000; ab181602; Abcam) and EZH2 (1:1000; ab186006; Abcam) for incubation overnight at 4°C. Later these films were incubated with goat anti-rabbit IgG H&L (HRP) secondary antibody (1:5000; ab205718; Abcam). Finally, ECL kits were applied to detect immunoreactivity.

### 2.7 Flow cytometry for apoptosis detection

Apoptosis was detected via AV-FITC/PI apoptosis detection kit (BD Biosciences, U.S.). After transfection, HT-29 and HCT-8 cells were harvested to resuspend in binding buffer and later double stained with 5 μL of AV-FITC and 10 μL of PI reagent for 15 minutes in the dark. At last, apoptotic cells (AV-FITC+) were monitored via flow cytometry (BD Biosciences).

### 2.8 Transwell

Transwell experiment was performed according to the previous method [30]. After the migration assay via Transwell, HT-29 and HCT-8 cells were suspended (1 × 105) in serum-free medium that was placed in upper chambers, and added complete medium to the bottom. After 48-hour incubation, the cells transferred to PVDF films were fixed with 4% paraformaldehyde to stain with crystal violet, observe and count under microscopes (magnification: ×100). For cell invasion, CRC cell invasion ability received evaluation through seeding 5 × 105 cells on upper chambers pre-coated with Matrigel (BD Biosciences). The other steps were the same in migration determination.

### 2.9 Double luciferase reporter gene experiment

Via luciferase reporter gene detection system (Promega, U.S.) to perform luciferase activity detection in reference to the instructions, WT and MUT 3ʹUTR fragments of EZH2 gene were simply cloned into pGL3 luciferase reporter vector (Promega); HT-29 and HCT-8 cells (2 × 104 cells/mL) were seeded into 6-well plates to culture with lipofectamine 3000 for 16 hours, and later co-transfected with reporter plasmids and miR-506-3p mimic; luciferase activity was detected via dual luciferase assay kit (PROMEGA) 72 hours after transfection.

### 2.10 Statistical methods

All experiments in triplicate, our team conducted statistical analysis via SPSS 22.0 software, data expressed as mean ± standard deviation (SD). Unpaired Student’s t test was employed for intergroup comparison, while one-way analysis of variance (ANOVA) was appllied for data comparison among multiple groups. * *P* < 0.05 means statistical significance. Graphpad prism 8.0 (Graphpad software inc., San Diego, USA) was used for data visualization.

## 3. Results

### 3.1 miR-506-3p was down-regulated, and EZH2 up-regulated in CRC

**Figure 1.**
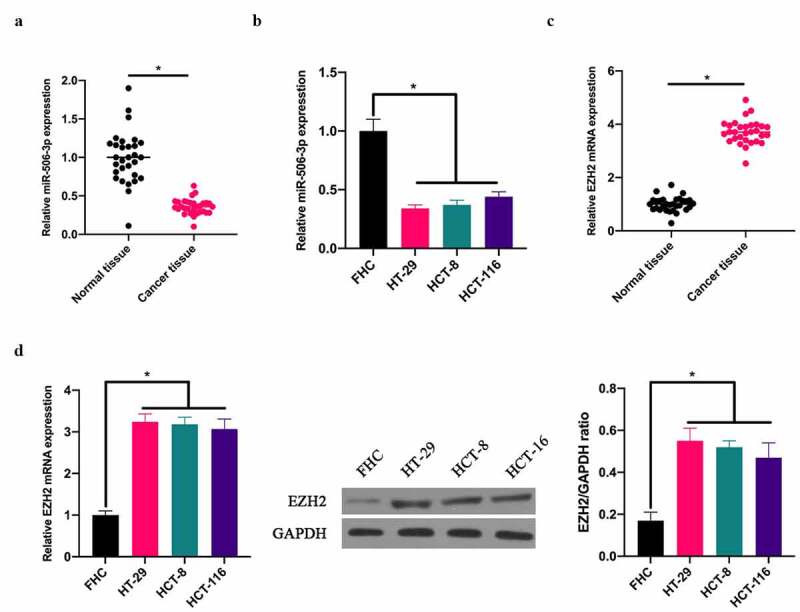
**miR-506-3p was down-regulated, and EZH2 up-regulated in CRC**. A: RT-qPCR to detect miR-506-3p in CRC tissues and adjacent tissues; B: RT-qPCR to detect miR-506-3p level in normal human colorectal epithelial cell FHC and CRC cell lines (HCT-116, HT-29 and HCT-8); C: RT-qPCR to detect EZH2 mRNA expression in CRC tissues and adjacent tissues; B: RT-qPCR and western blot to detect EZH2 mRNA and protein levels in normal colorectal epithelial cell FHC and CRC cell lines (HCT −116, HT-29 and HCT-8), data expressed as mean ± SD (n = 3), *P < 0.05

To exploit the role of miR-506-3p in CRC, our team applied RT-qPCR to detect miR-506-3p expression in CRC tissues and adjacent normal tissues. miR-506-3p expression in CRC tissues, as well as in HT-29, HCT-8, and HCT116 cell lines was lower than that in normal adjacent tissues and colorectal epithelial cells FHC (Figure 1a & 1b). Additionally, RT-qRCR and western blot assays revealed that EZH2 expression in CRC tissues and colon cancer cell lines HT-29, HCT-8, HCT116 was higher than that in normal adjacent tissues as well as colorectal epithelial cells FHC (Figure 1c & 1d).

### 3.2 Overexpressing miR-506-3p inhibited CRC development

**Figure 2.**
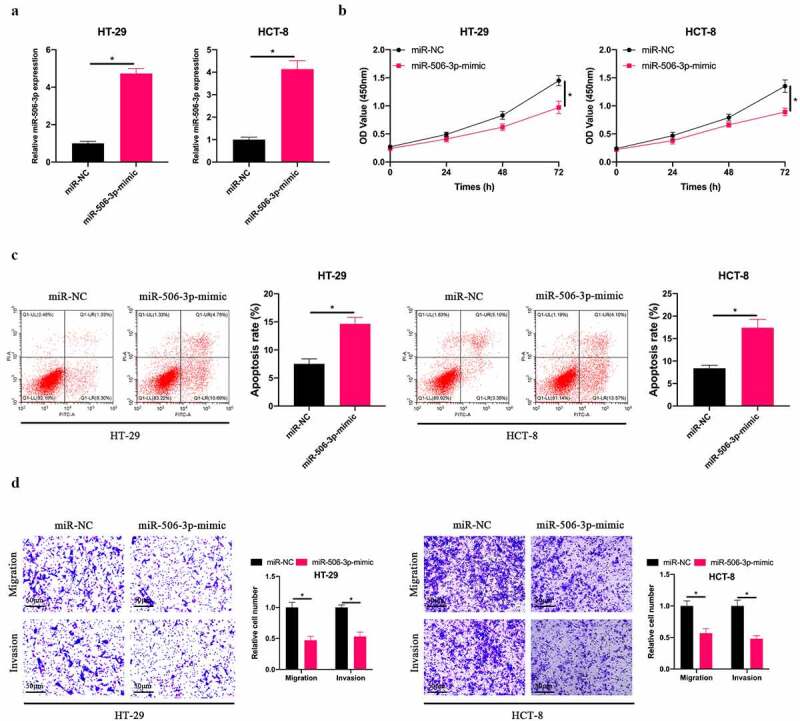
**Overexpressing miR-506-3p inhibited CRC development**. A: RT-qPCR to detect miR-506-3p expression in HT-29 and HCT-8 cells after transfecting miR-506-3p-mimic; B: CCK-8 to detect HT-29 and HCT-8 cell proliferation after transfecting miR-506-3p-mimic; C: Flow cytometry to detect HT-29 and HCT-8 cell apoptosis after transfecting miR-506-3p-mimic; D: Transwell to detect HT-29 and HCT-8 cells invasion and migration after transfecting miR-506-3p-mimic, data represented by mean ± SD (n = 3), *P < 0.05

Subsequently, our team selected HT-29 and HCT-8 cells with the lowest miR-506-3p expression for follow-up experiments, up-regulated miR-506-3p level in HT-29 and HCT-8 cells (Figure 2a), and examined the impact of up-regulating miR-506-3p on CRC cell biological progression. Overexpressing miR-506-3p signally inhibited HT-29 and HCT-8 cell proliferation (Figure 2b). Additionally, after up-regulating miR-506-3p, HT-29 and HCT-8 cell apoptotic rate elevated visually (Figure 2c). At the same time, up-regulating miR-506-3p also inhibited HT-29 and HCT-8 cell invasion and migration (Figure 2d). These findings indicate that miR-506-3p is under-expressed in CRC. Overexpressing miR-506-3p is available to inhibit CRC development.

### 3.3 Downregulation EZH2 inhibited CRC development

**Figure 3.**
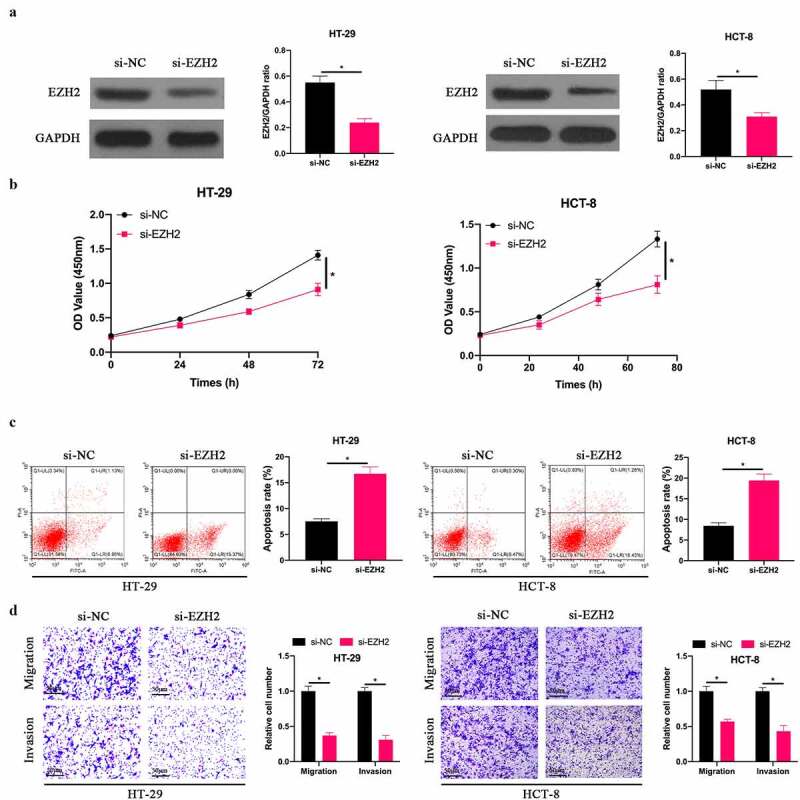
**Silencing EZH2 inhibited CRC development**. A: Western blot to detect EZH2 expression in HT-29 and HCT-8 cells after transfecting si-EZH2; B: CCK-8 to detect HT-29 and HCT-8 cell proliferation after transfecting si-EZH2; C: Flow cytometry to detect HT-29 and HCT-8 cell apoptosis after transfecting si-EZH2; D: Transwell to detect HT-29 and HCT-8 cell invasion and migration after transfecting si-EZH2, data represented by mean ± SD (n = 3), *P < 0.05

In order to explore the effect of EZH2 on the biological function of CRC, we silenced EZH2 in HT-29 and HCT-8 cells (Figure 3a). The findings revealed (Figure 3b-d) that after silencing EZH2, HT-29 and HCT-8 cell proliferation was signally inhibited, the apoptotic rate was signally elevated, and invasion and migration in quantity were signally reduced. This indicates that EZH2 is up-regulated in CRC, and silencing EZH2 is available to inhibit CRC biological progression.

## 3.4 miR-506-3p targeted EZH2 in CRC

**Figure 4.**
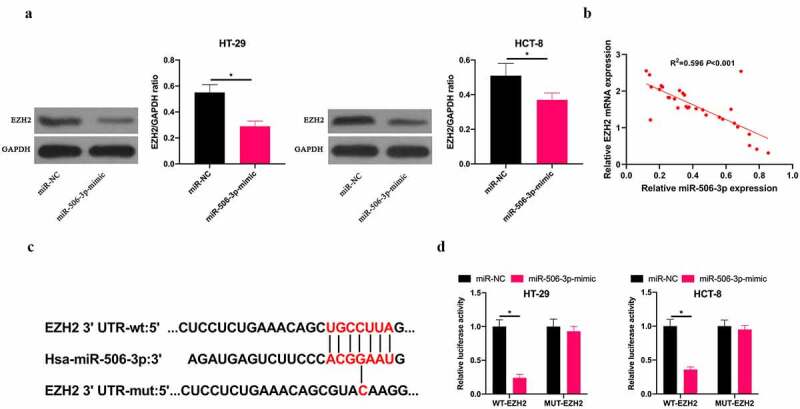
**miR-506-3p targeted EZH2 in CRC**. A: Western blot to detect EZH2 expression in HT-29 and HCT-8 cells after transfecting miR-506-3p-mimic; B: Correlation analysis of miR-506-3p and EZH2 in tissues of CRC patients; C: Targeted binding sites of miR-506-3p and EZH2; D: Dual luciferase report experiment to verify the targeting relationship between miR-506-3p and EZH2, data expressed as mean ±SD (n = 3), *P < 0.05

Next, we exploited the relationship between miR-506-3p and EZH2 in CRC. We found that after overexpressing miR-506-3p, EZH2 level in HT-29 and HCT-8 cells was obviously reduced (Figure 4a). Additionally, miR-506-3p and EZH2 expression levels in CRC tissues were negatively correlated (Figure 4b). Therefore, we speculate that miR-506-3p and EZH2 have a targeting relationship. Via miRDB database (http://www.microrna.org/microrna/home.do), our team predicted that EZH2 3ʹ-UTR has binding sites with miR-506-3p (Figure 4c). Aiming to further confirm the prediction findings, our team constructed WT EZH2 and MUT 3ʹ-UTR luciferase reporter vectors. At the same time, we transfected HT-29 and HCT-8 cells with miR-506-3p, NC miRNA, and luciferase reporter vector, respectively. The findings revealed that WT EZH2 signally weakened luciferase activity in miR-506-3p-mimic group, while MUT EZH2 had no impact on it (Figure 4d). These findings indicate that miR-506-3p targets EZH2 level in CRC.

## 3.5 miR-506-3p affected CRC progress through regulating EZH2

**Figure 5.**
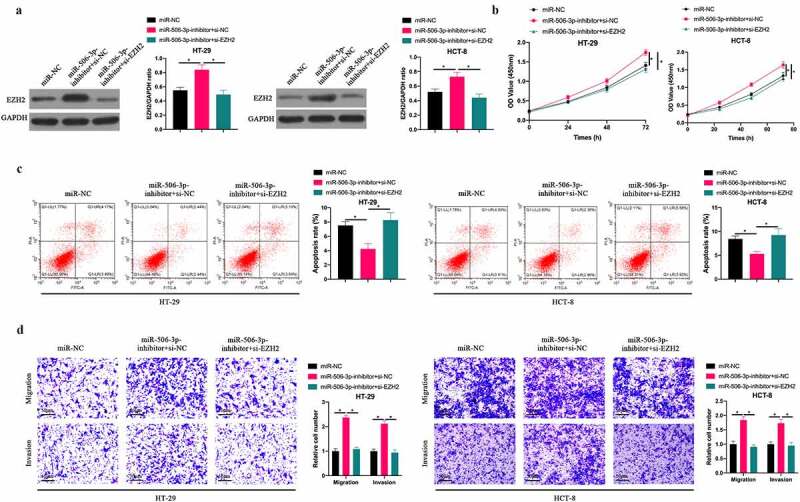
**miR-506-3p inhibited CRC development through regulating EZH2**. A: Western blot to detect EZH2 expression after co-transfecting miR-506-3p-inhibitor and si-EZH2; B: CCK-8 to detect HT- 29 and HCT-8 cell proliferation after co-transfecting miR-506-3p-inhibitor and si-EZH2; C: Flow cytometry to detect HT-29 and HCT-8 cell apoptosis after co-transfecting miR-506-3p-inhibitor and si-EZH2; D: Transwell to detect HT-29 and HCT-8 cell invasion and migration after co-transfecting miR −506-3p-inhibitor and si-EZH2, data represented by mean ± SD (n = 3), *P < 0.05

To probe into whether miR-506-3p inhibits CRC progression through regulating EZH2, we suppressed EZH2 while silencing miR-506-3p. After silencing miR-506-3p, EZH2 level in HT-29 and HCT-8 cells increased signally, whereas EZH2 level was reversed after co-transfecting si-EZH2 (Figure 5a). Subsequently, CRC biological progress experiment findings revealed that silencing miR-506-3p visually promoted HT-29 and HCT-8 cell proliferation, invasion and migration, and inhibited apoptosis. After co-transfecting si-EZH2, the impact of silencing miR-506-3p on HT-29 and HCT-8 cells was obviously reversed. In conclusion, this indicates that miR-506-3p is available to target EZH2 to regulate CRC progression.

## 4. Discussion

At present, except for a small number of CRC patients with obvious familial diseases, most of them are sporadic. CRC pathogenesis is related to individual gene mutations, like chromosomal mutations and DNA mismatch repair [5,31,32]. In addition to primary tumors, CRC infiltration and metastasis seriously endanger human health. Local invasion is the most universal form of CRC invasion. The tumor gradually infiltrates the surrounding tissues or organs through planting and metastasis, resulting in corresponding clinical symptoms [33,34]. However, the pathogenesis of CRC has not yet been fully elucidated. In this study, we found that miR-506-3p can bind to the 3ʹUTR of EZH2 to inhibit EZH2 expression, thereby preventing the malignant behavior of CRC.

miRNAs are non-coding RNAs (18–25 nt) in human genes, accounting for only 1% [35,36], but they are available to regulate over 1/3 gene expression, modification, translation and transcription [37,38]. Based on previous studies, miRNAs are widely involved in tumor occurrence and development through targeting oncogenes or tumor suppressor genes. Dysregulated miRNAs can result in a series of cascade reactions and feedback pathways involving multiple mRNAs and target genes. At the same time, multiple miRNAs are available to target the same gene, thereby regulating the gene expression [39–41]. Therefore, the miRNA network is very complicated. Many studies have shown that miR-506-3p acts as a tumor suppressor gene in cancers, including osteosarcoma [42], glioma [43], nasopharyngeal carcinoma [44] and so on. In our work, we found that overexpressing miR-506-3p can inhibit the proliferation, migration and invasion of CRC cells and promote apoptosis. These data support that miR-506-3p act as a tumor suppressor gene in CRC, consistent with that in other cancers. Although the same miRNA has similar functions in different cancer tissues, the clinical effects of the same miRNA regulation are inconsistent. Therefore, it is necessary to conduct clinical trials targeting miR-506-3p in the future to explore the therapeutic effects of miR-506-3p in different cancers. A recent study reported that miR-506-3p expression can increase the cisplatin sensitivity of osteosarcoma [45]. We speculate that miR-506-3p also has a similar role in cisplatin resistance in CRC, which can be explored in the next step. Previous studies have shown that miR-506-3p is competitively bound by LncRNA or circRNA, thereby promoting the expressions of downstream target genes [46,47]. Due to limited conditions, this study did not explore the upstream regulatory factors of miR-506-3p. However, in future research, it is necessary to have a deeper and more comprehensive understanding of the up-regulatory factors and downstream targets of miR-506-3p, as well as the regulatory network of miR-506-3p in cancer is of great significance in the prevention and treatment of cancer.

For EZH2 is over-expressed in various cancers, inhibitors against EZH2 have also been developed. Sitaxentan, an EZH2 inhibitor, has been approved for treating epithelioid sarcoma as the first FDA-approved EZH2 inhibitor [78]. In reference to reports, other EZH2 inhibitors like GSK343 and GSK236 are available to inhibit the tumor progression of various cancers as well, like glioblastoma [48], head and neck cancer [49] and breast cancer [50]. In this study, miR-506-3p posed targeted inhibition on EZH2 expression in CRC, and knocking down EZH2 could reverse the promotion of miR-506-3p inhibitor on the malignant behavior of CRC. This further supports the results of previous studies [51–54]. Recent studies have shown that competitive EZH2 inhibitors can exhibit anti-cancer activity in a variety of cancers, such as 3-deazaneplanocin A [55]. However, EZH2 inhibitors cannot completely inhibit the oncogenic activity of EZH2 in SWI/SNF mutant cancers [56]. Therefore, it is necessary to develop new EZH2 inhibitors to block the catalytic and non-catalytic activities of EZH2.

## 5. Conclusion

Our results indicate that mir-506-3P can reduce EZH2 expression in CRC cells by binding to the 3ʹUTR end of EZH2, thereby preventing CRC proliferation, invasion and migration. This provides a new potential target for the clinical treatment of CRC in the future.

## Highlights

1. miR-506-3p is under-expressed and EZH2 is highly expressed in colorectal cancer.
2. Overexpressing miR-506-3p or knocking down EZH2 can inhibit colorectal cancer progress.
3. 3. MiR-506-3p targets and regulates EZH2 expression.
4. 4. The promotion effect of inhibiting miR-506-3p on the malignant behavior of colorectal cancer is reversed by knocking down EZH2.
